# Evaluation of the ability of the trypsin-like peptidase activity assay to detect severe periodontitis

**DOI:** 10.1371/journal.pone.0256538

**Published:** 2021-08-20

**Authors:** Masanori Iwasaki, Michihiko Usui, Wataru Ariyoshi, Keisuke Nakashima, Yoshie Nagai-Yoshioka, Maki Inoue, Kaoru Kobayashi, Tatsuji Nishihara

**Affiliations:** 1 Tokyo Metropolitan Institute of Gerontology, Tokyo, Japan; 2 Division of Periodontology, Kyushu Dental University, Kitakyushu, Japan; 3 Division of Infections and Molecular Biology, Kyushu Dental University, Kitakyushu, Japan; 4 Endowed Course, Periodontal Medicine, Kyushu Dental University, Kitakyushu, Japan; 5 Graduate School of Dentistry, Kyushu Dental University, Kitakyushu, Japan; University of California, Davis, UNITED STATES

## Abstract

**Objectives:**

N-benzoyl-DL-arginine peptidase (trypsin-like peptidase) is specifically produced by certain strains of periodontitis-associated bacteria. We aimed to examine the effectiveness of an objectively quantified trypsin-like peptidase activity assay (TLP-AA) for detecting severe periodontitis.

**Methods:**

The study population included 347 adults (108 men and 239 women; average age, 43.3 years) who underwent a full-mouth periodontal examination. Specimens for the TLP-AA were obtained using tongue swabs. Using a color reader, the TLP-AA results were obtained as a* values, with higher positive a* values indicating an increased intense enzymatic activity. The predictive validity of the TLP-AA results for severe periodontitis was assessed using receiver operating characteristic curve analysis and the periodontitis case definition provided by the Centers for Disease Control and Prevention/American Academy of Periodontology as the gold standard. Furthermore, multivariable logistic regression analyses were performed to predict severe periodontitis using the TLP-AA results and health characteristics, as the exposure variables.

**Results:**

Severe periodontitis was observed in 5.2% of the participants. TLP-AA had high diagnostic accuracy for severe periodontitis, with an area under the curve of 0.83 (95% confidence interval [CI]: 0.75–0.92). The cut-off score for the a* value that best differentiated individuals with severe periodontitis was 0.09, with a sensitivity of 83% and specificity of 77%. Multivariable logistic regression analyses revealed that the TLP-AA results were significantly associated with severe periodontitis after adjusting for health characteristics (adjusted odds ratios: 1.90 [95% CI: 1.37–2.62] for the a* value).

**Conclusions:**

Objectively quantified TLP-AA results are potentially useful for detecting severe periodontitis in epidemiological surveillance.

## Introduction

Periodontitis, particularly severe periodontitis, is associated with serious adverse health events, including cardiovascular disease and death [1, 2]. Thus, epidemiological surveillance of periodontitis is important [3]. The performance of clinical examinations is the standard and preferred approach for periodontitis surveillance. However, the examination requires several resources, including time, equipment, and manpower (trained examiners). In addition, the clinical examination may place individuals at risk of bacteremia development [4]. Thus, the surveillance of periodontitis at the population level remains challenging. This is mirrored by the current situation in Japan, where periodontitis surveillance at the community level or workplace is not performed frequently [5, 6]. Furthermore, according to a recent survey conducted by the Japan Dental Association [7], approximately 70% of the survey participants reported that they did not visit the dentist regularly, indicating that many individuals do not undergo periodontal health surveillance.

Recently, we developed a less invasive and more readily applicable method of detecting periodontitis, based on the measurement of the activity of N-benzoyl-DL-arginine peptidase (trypsin-like peptidase) in tongue swabs. Trypsin-like peptidase is specifically produced by certain strains of *Porphyromonas gingivalis*, *Tannerella forsythia*, *Treponema denticola*, and *Capnocytophaga ochracea* [8, 9]. Among these enzymes, gingipains, the main component of the trypsin-like peptidase derived from *Porphyromonas gingivalis*, were reported to induce the apoptosis of fibroblasts [10] and epithelial cells [11, 12], and play important roles in the onset and progression of periodontitis via interaction with the host immune response. Furthermore, a recent study revealed that trypsin-like peptidases from periodontopathic bacteria rapidly degraded the adhesive extracellular matrix produced by the junctional epithelium of the gingiva [13]. The method used in our preliminary study had high diagnostic accuracy for severe periodontitis [14]. However, a definitive conclusion could not be extracted owing to the small sample size. In addition, the activity of trypsin-like peptidase was scored visually; therefore, it was prone to inter- and intra-rater variability.

Moreover, we examined the effectiveness of an objectively quantified trypsin-like peptidase activity assay (TLP-AA) in detecting severe periodontitis in a study with an adequate sample size.

## Materials and methods

### Study design, setting, and participants

The study population comprised a cohort of workers at the Nishinihon Occupational Health Service Center in Fukuoka, Japan. Individuals who participated in an annual health check-up at the workplace were invited to participate in this study.

The inclusion criteria were as follows: at least two teeth present, age ≥20 years, and ability to read and understand Japanese. The exclusion criteria were as follows: antibiotic usage within 1 month prior to the study, administration of periodontal treatment within 1 month prior to the study; and pre-diagnosis of a severe or terminal disease, such as advanced heart failure, end-stage kidney disease, or advanced-stage cancer.

All the study participants provided written informed consent. This study was conducted in full accordance with the ethical principles of the Declaration of Helsinki and was approved by the Ethics Committee of Kyushu Dental University (Approval number: 19–32).

The sample size calculation was performed using R version 4.0.2 (R Foundation for Statistical Computing, Vienna, Austria). Considering the prevalence of severe periodontitis in our preliminary study (4.8%) [14], the estimated sample size was 329, based on an area under the curve (AUC) of 0.80, corresponding to a one-sided alpha of 0.01, power of 95%, and attrition rate of 3%.

### Data collection

#### Trypsin-like peptidase activity assay

Before the oral health examination, one examiner who was blinded to the periodontal status of the participants performed the TLP-AA using a TLP-AA kit (ADTEC Co., Ltd., Oita, Japan) and FD-7 Color Reader (Konica Minolta Holdings, Inc., Tokyo, Japan).

Tongue swabs collected from the participants were used as specimens for the assay. The participants were asked to refrain from eating, smoking, tongue cleaning, tooth brushing, and mouth wash usage for 1 hour prior to the examination to meet the specimen collection condition.

The TLP-AA was performed according to the manufacturer’s instructions. Briefly, the tongue swab was pressed onto a test plate disc containing N-benzoyl-arginine β-naphthylamide (N α-Benzoyl DL-arginine β-naphthylamide hydrochloride) matrix (S1A Fig). If the relevant enzymes (i.e., N-benzoyl-DL-arginine peptidase) were present in the tongue swab, the matrix was broken down by enzymes, and β-naphthylamide was released. Subsequently, the 4-(dimethylamino)-cinnamaldehyde color developer was placed onto the test plate; any released β-naphthylamide reacted with the developer, and a red-colored chemical compound was produced (S1B Fig). Consequently, the TLP-AA-Kit was used to macroscopically determine the pigment changes on the matrix disc and examine whether any N-benzoyl-DL-arginine peptidase activation occurred in the specimen.

The intensity of the matrix disc color was assessed by two means: using the color reader and by visual inspection, as described below.

1. Using an FD-7 Color Reader, the a* value of the matrix disc color was measured (S1C Fig). Based on the L* a* b* color system developed by the International Commission on Illumination, the a* value represents perception on the red/magenta-green spectrum. The a* value reflects an objectively scored result of the TLP-AA and was set as a primary indicator in the diagnostic ability assessment.
2. Using the score interpretation sample, the intensity score of the matrix disc color was visually ranked across five score values in 0.5 units (range, 1–5). The visual inspection value reflects a subjectively scored result of the TLP-AA.

In both methods, a higher measurement score (i.e., higher positive a*value and higher visual inspection value) indicated a stronger red color in the matrix disc and a more intense enzymatic activity (i.e., higher trypsin concentration) in the sample.

#### Oral health examinations

Twelve qualified dentists who were blinded to the results of the TLP-AA conducted the oral health examinations, which involved the following: determining the number of teeth and presence of dentures using sufficient artificial illumination and recording the periodontal probing depth (PPD), gingival recession, and bleeding on probing at six sites (mesio-buccal, mid-buccal, disto-buccal, mesio-lingual, mid-lingual, and disto-lingual) for every tooth except for the third molars, by using a graduated periodontal probe (Williams COLORVUE® Probe, Hu-Friedy Mfg. Co., LLC., Frankfurt, Germany) and a mouth mirror (HD MIRRORS, Hu-Friedy, Chicago, IL, USA). Then, the clinical attachment loss (CAL) was calculated using the PPD and gingival recession.

Prior to the examinations, calibrations were performed at Kyushu Dental University using volunteer patients. Eleven examiners obtained a minimum kappa value of 0.8, relative to the gold standard obtained by another examiner (M.U.). All examiners obtained intra-examiner kappa values >0.8 for both PPD and gingival recession. For the kappa calculations, PPD and gingival recession values that were exactly equal to or had a difference within 1 mm were considered to indicate agreement.

The oral hygiene and tongue-coating statuses were also assessed during the examination. The oral hygiene status was assessed using the simplified oral hygiene index (OHI-S) [15]. The range of the OHI-S is 0–6, with higher scores indicating a poorer oral hygiene status. The tongue-coating status was assessed using the tongue-coating index (TCI) [16]. The range of the TCI is 0–100, with higher scores indicating a larger amount of tongue coating.

#### Other characteristics

Data on age, sex, smoking status, drinking behavior, physical activity level, and diabetes status were collected through self-administered questionnaires. Data on height, weight, and serum glycated hemoglobin A1c (HbA1c) levels were obtained from the health check-up records. The smoking status was dichotomized into current smoker or not. The frequency and amount of alcohol consumed were queried; according to the second phase of Healthy Japan 21 [17] and related studies [18, 19], high-risk drinking was defined as an answer of “every day” or “sometimes” to the question, “how often do you drink: every day, sometimes, or rarely” and an answer of “two drinks or more” to the question “how many drinks containing alcohol do you have on a typical day when drinking?” A low physical activity level was defined as an answer of “no” to the question, “in your daily life do you walk or do any equivalent amount of physical activity for more than 1 hour a day?” Diabetes was defined as a self-reported physician’s diagnosis, self-reported use of insulin or other glucose-lowering drugs, and/or an HbA1c level ≥6.5%. The body mass index (BMI) was calculated as follows: BMI = height/(weight)^2^. Overweight was defined as a BMI value ≥25 kg/m^2^.

### Statistical analyses

The TLP-AA results were evaluated against a periodontal diagnosis based on the six-site full-mouth periodontal examination (FMPE). The periodontitis case definition provided by the Centers for Disease Control and Prevention/American Academy of Periodontology (CDC/AAP) [20] served as the gold standard for determining the predictive validity of the TLP-AA. In accordance with the CDC/AAP definition, severe periodontitis was defined as having ≥2 interproximal sites with a CAL ≥6 (not on same tooth) and ≥1 mm interproximal site with a PPD ≥5 mm. Student’s *t*-test, the Mann-Whitney *U* test, and the chi-squared test were used (as appropriate) to compare the health characteristics between the groups with and without severe periodontitis. The Shapiro–Wilk test was used to determine whether continuous variables were normally distributed.

Correlations between the a* value, visual inspection value, and oral health status variables were examined using Spearman’s rank correlation coefficient.

The predictive validity of the TLP-AA results (both a* and visual inspection values) for severe periodontitis was assessed using receiver operating characteristic (ROC) curve analysis. The AUC was estimated and interpreted as follows: AUC >0.8, high predictive power; 0.8 ≥AUC >0.7, useful predictive power; and AUC ≤0.7, low predictive power [3]. The optimal cut-off thresholds for identifying individuals with severe periodontitis were determined using the highest Youden’s Index (sensitivity + specificity − 1).

Furthermore, we performed multivariable logistic regression analyses to predict severe periodontitis using the TLP-AA results and the health characteristics as exposure variables. Three models were constructed. Model 1 included the TLP-AA results. Model 2 included the TLP-AA results and the demographic and health-related variables (full model). Model 3 included the best significant subset that was selected from the full model. The predictive validity of the models was assessed by calculating the AUC. We also calculated the sensitivity, specificity, and Bayesian information criterion. The sensitivity and specificity were based on the dichotomized classification of the predicted probability of being a case at a selected cut-off point that was as close as possible to the predicted prevalence of the outcome.

As a sensitivity analysis, the performance of the TLP-AA in detecting severe periodontitis was assessed using the AAP/European Federation of Periodontology (EFP) case definition [21]. In accordance with the AAP/EFP definition, severe periodontitis was defined as the presence of an interdental CAL ≥5 mm in two or more nonadjacent teeth or the presence of buccal or oral CAL ≥3 mm with a PPD >3 mm in two or more teeth and interdental CAL at the site of greatest loss ≥5 mm [22].

Statistical analyses were performed using Stata version 16.1 (Stata Corporation LP, College Station, TX, USA), with the level of significance (two-tailed) set at 0.05.

## Results

In total, 986 individuals who underwent a medical check-up and met the inclusion/exclusion criteria agreed to participate in our study. Of these, 408 met the sample collection condition for the TLP-AA and underwent an oral health examination, completed the questionnaires, and provided tongue swabs. In total, 61 out of 408 adults had missing data and were excluded from the analysis. Thus, the study population comprised 347 adults (108 men and 239 women; average age, 43.3 years).

Based on the CDC/AAP and AAP/EFP definitions, severe periodontitis was observed in 18 (prevalence, 5.2%) and 52 participants (prevalence, 15.0%), respectively. Table 1 shows the TLP-AA results according to the presence of severe periodontitis. The mean (standard deviation) of the a* value and median (interquartile range) of the visual inspection score in the total study population were -0.4 (1.4) and 1.0 (1.0–1.5), respectively.

**Table 1:** Characteristics of the study population according to periodontitis classifications.

	Total	CDC/AAP definition			AAP/EFP definition		
		Not severe	Severe		Not severe	Severe	
	*n* = 347	*n* = 329	*n* = 18	*p* *	*n* = 295	*n* = 52	*p* *
Results of the TLP-AA							
a* value*	-0.4 (1.4)	-0.5 (1.2)	1.7 (2.7)	<0.01	-0.6 (1.1)	0.9 (2.2)	<0.01
Visual inspection score^†^	1.0 (1.0–1.5)	1.0 (1.0–1.5)	1.8 (1.5–2.0)	<0.01	1.0 (1.0–1.0)	1.5 (1.0–2.0)	<0.01
Oral health variables							
Number of teeth^†^	28 (27–28)	28 (27–28)	26 (24–27)	<0.01	28 (27–28)	27 (25–28)	<0.01
Average PPD (mm)^†^	1.7 (1.5–2.0)	1.7 (1.5–1.9)	2.5 (2.2–2.9)	<0.01	1.7 (1.5–1.9)	2.2 (2.0–2.4)	<0.01
Percentage of sites with PPD ≥3 mm^†^	7.7 (3.3–18.5)	7.4 (3.1–15.5)	38.7 (28.6–57.3)	<0.01	6.5 (3.0–13.1)	26.8 (18.6–41.7)	<0.01
Percentage of sites with PPD ≥4 mm^†^	0 (0–1.5)	0 (0–1.2)	12.6 (8.9–18.7)	<0.01	0 (0–0.6)	5.5 (2.4–12.1)	<0.01
Percentage of sites with PPD ≥5 mm^†^	0 (0–0.6)	0 (0–0)	7.3 (5.1–11.3)	<0.01	0 (0–0)	2.4 (1.2–6.3)	<0.01
Percentage of sites with PPD ≥6 mm^†^	0 (0–0)	0 (0–0)	2.7 (1.2–4.2)	<0.01	0 (0–0)	1.2 (0–2.3)	<0.01
Percentage of teeth with PPD ≥3 mm^†^	28.6 (11.5–53.6)	28.6 (11.1–46.4)	75.5 (60.7–88.0)	<0.01	25.0 (10.7–41.7)	65.9 (50.0–79.6)	<0.01
Percentage of teeth with PPD ≥4 mm^†^	0 (0–7.1)	0 (0–7.1)	41.4 (33.3–50.0)	<0.01	0 (0–3.7)	20.4 (10.7–37.7)	<0.01
Percentage of teeth with PPD ≥5 mm^†^	0 (0–3.6)	0 (0–0)	25.0 (17.9–32.0)	<0.01	0 (0–0)	10.9 (6.1–19.6)	<0.01
Percentage of teeth with PPD ≥6 mm^†^	0 (0–0)	0 (0–0)	9.5 (7.4–15.4)	<0.01	0 (0–0)	3.7 (0–7.7)	<0.01
Average CAL (mm)^†^	1.8 (1.6–2.0)	1.8 (1.6–2.0)	2.7 (2.5–3.3)	<0.01	1.7 (1.5–1.9)	2.4 (2.1–2.7)	<0.01
Percentage of sites with CAL ≥3 mm^†^	11.9 (5.4–22.4)	11.1 (4.9–19.8)	47.5 (35.4–73.8)	<0.01	10.0 (4.8–17.9)	34.7 (22.0–50.5)	<0.01
Percentage of sites with CAL ≥4 mm^†^	0.6 (0–2.6)	0.6 (0–2.4)	19.1 (12.8–28.0)	<0.01	0.6 (0–1.4)	9.0 (5.3–19.0)	<0.01
Percentage of sites with CAL ≥5 mm^†^	0 (0–0.6)	0 (0–0.6)	10.0 (6.2–13.2)	<0.01	0 (0–0)	3.9 (2.2–7.8)	<0.01
Percentage of sites with CAL ≥6 mm^†^	0 (0–0)	0 (0–0)	4.6 (2.5–6.8)	<0.01	0 (0–0)	1.2 (0–3.0)	<0.01
Percentage of teeth with CAL ≥3 mm^†^	35.7 (21.4–57.1)	35.7 (17.9–53.8)	80.2 (69.2–95.5)	<0.01	32.1 (17.9–50.0)	69.4 (55.8–85.7)	<0.01
Percentage of teeth with CAL ≥4 mm^†^	3.6 (0–11.1)	3.6 (0–10.7)	48.9 (37.0–68.0)	<0.01	3.6 (0–7.4)	31.5 (17.3–50.0)	<0.01
Percentage of teeth with CAL ≥5 mm^†^	0 (0–3.6)	0 (0–3.6)	28.6 (19.2–37.5)	<0.01	0 (0–0)	14.8 (7.3–26.8)	<0.01
Percentage of teeth with CAL ≥6 mm^†^	0 (0–0)	0 (0–0)	14.6 (11.1–20.0)	<0.01	0 (0–0)	3.9 (0–11.8)	<0.01
BOP (%)^†^	6.0 (1.8–12.5)	5.4 (1.2–11.9)	19.6 (10.7–26.2)	<0.01	4.2 (1.2–10.7)	12.2 (6.5–23.5)	<0.01
OHI-S*	2.0 (0.2)	2.0 (0.2)	2.2 (0.4)	0.01	2.0 (0.2)	2.1 (0.3)	<0.01
TCI (%)*	49.4 (5.7)	49.4 (5.8)	50.3 (3.6)	0.50	49.2 (6.1)	50.4 (2.7)	0.17
Denture use^‡^	3 (0.9%)	3 (0.9%)	0 (0%)	0.68	1 (0.3%)	2 (3.8%)	0.01
Tooth brushing ≥2 times/day^‡^	320 (92.2%)	304 (92.4%)	16 (88.9%)	0.59	272 (92.2%)	48 (92.3%)	0.98
Interdental cleaning device use^‡^	169 (48.7%)	160 (48.6%)	9 (50.0%)	0.91	143 (48.5%)	26 (50.0%)	0.84
Regular dental check-ups^‡^	141 (40.6%)	133 (40.4%)	8 (44.4%)	0.74	115 (39.0%)	26 (50.0%)	0.14
Other variables							
Age*	43.3 (12.2)	42.6 (11.9)	55.7 (10.7)	<0.01	41.8 (11.8)	51.8 (11.4)	<0.01
Sex^‡^				0.46			0.06
Women	239 (68.9%)	228 (69.3%)	11 (61.1%)		209 (70.8%)	30 (57.7%)	
Men	108 (31.1%)	101 (30.7%)	7 (38.9%)		86 (29.2%)	22 (42.3%)	
High-risk drinking^‡^	30 (8.6%)	26 (7.9%)	4 (22.2%)	0.04	22 (7.5%)	8 (15.4%)	0.06
Current smoker^‡^	36 (10.4%)	34 (10.3%)	2 (11.1%)	0.92	29 (9.8%)	7 (13.5%)	0.43
Low physical activity level^‡^	195 (56.2%)	185 (56.2%)	10 (55.6%)	0.96	166 (56.3%)	29 (55.8%)	0.95
Overweight^‡^	62 (17.9%)	55 (16.7%)	7 (38.9%)	0.02	48 (16.3%)	14 (26.9%)	0.06
Diabetes^‡^	10 (2.9%)	9 (2.7%)	1 (5.6%)	0.49	8 (2.7%)	2 (3.8%)	0.65

*presented as mean (SD)

^†^presented as median (IQR)

^‡^presented as N (%)

AAP, American Academy of Periodontology; BOP, bleeding on probing; CAL, clinical attachment loss; CDC, Centers for Disease Control and Prevention; EFP, European Federation of Periodontology; IQR, interquartile range; OHI-S, simplified oral hygiene index; PPD, periodontal probing depth; SD, standard deviation; TCI, tongue-coating index; TLP-AA, trypsin-like peptidase activity assay

Table 1 also shows the health characteristics according to the presence of severe periodontitis. The participants with severe periodontitis (based on the two definitions) were associated with poor periodontal health parameters and a poor oral hygiene status. Those with CDC/AAP severe periodontitis were older and had a higher proportion of high-risk drinking and overweight status than those without severe periodontitis. The participants with AAP/EFP severe periodontitis were older and had a higher proportion of denture use than those without severe periodontitis.

Table 2 shows the correlations between the a* value, visual inspection score, and oral health status variables. The a* value and the visual inspection score were significantly and positively correlated (Spearman’s rank correlation coefficient [ρ] = 0.70, *p*<0.05). Additionally, weak but significantly positively correlations of the a* value with all clinical periodontal parameters (ρ range, 0.14–0.23) and the OHI-S (ρ = 0.14, *p*<0.05) were found. In contrast, the correlations between the visual inspection score and percentage of teeth with a PPD ≥3 mm (ρ = 0.10, *p* = 0.05) and BOP (ρ = 0.09, *p* = 0.09) only showed a trend toward significance.

**Table 2:** Correlations among the a* value, visual inspection score, and oral health status variables.

	a* value	Visual inspection score
Visual inspection score	0.70*	-
Number of teeth	-0.01	-0.06
Average PPD (mm)	0.18*	0.10^†^
Percentage of sites with PPD ≥3 mm	0.19*	0.11*
Percentage of sites with PPD ≥4 mm	0.15*	0.18*
Percentage of sites with PPD ≥5 mm	0.21*	0.24*
Percentage of sites with PPD ≥6 mm	0.18*	0.24*
Percentage of teeth with PPD ≥3 mm	0.18*	0.10^†^
Percentage of teeth with PPD ≥4 mm	0.15*	0.17*
Percentage of teeth with PPD ≥5 mm	0.21*	0.23*
Percentage of teeth with PPD ≥6 mm	0.18*	0.24*
Average CAL (mm)	0.19*	0.14*
Percentage of sites with CAL ≥3 mm	0.21*	0.15*
Percentage of sites with CAL ≥4 mm	0.23*	0.23*
Percentage of sites with CAL ≥5 mm	0.22*	0.25*
Percentage of sites with CAL ≥6 mm	0.19*	0.24*
Percentage of teeth with CAL ≥3 mm	0.19*	0.13*
Percentage of teeth with CAL ≥4 mm	0.22*	0.21*
Percentage of teeth with CAL ≥5 mm	0.22*	0.25*
Percentage of teeth with CAL ≥6 mm	0.19*	0.24*
BOP (%)	0.14*	0.09^†^
OHI-S	0.14*	0.15*
TCI (%)	0.08	0.12*

Spearman’s Rho values are presented

*p<0.05,

^†^*p*<0.1

BOP, bleeding on probing; CAL, clinical attachment loss; OHI-S, simplified oral hygiene index; PPD, periodontal probing depth; TCI, tongue-coating index

**Fig 1:**
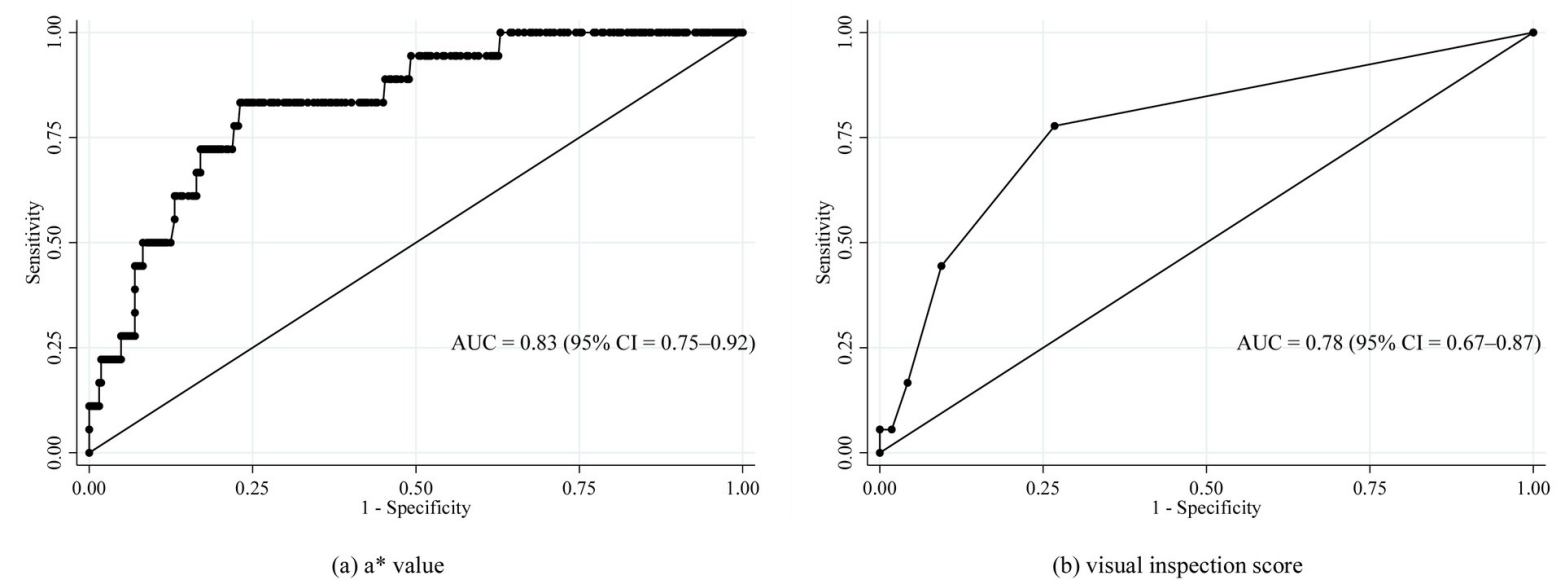
Receiver operating characteristic curves of TLP-AA results for severe periodontitis based on the CDC/AAP definition. The receiver operating characteristic curves of the TLP-AA results for severe periodontitis as defined by the CDC/AAP, are presented. AAP, American Academy of Periodontology; CDC, Centers for Disease Control and Prevention; TLP-AA, trypsin-like peptidase activity assay.

The ROC curves of the a* value and those of the visual inspection score for CDC/AAP severe periodontitis are presented in Fig 1. The diagnostic accuracy of the a* value for severe periodontitis was high, with an AUC of 0.83 (95% confidence interval [CI]: 0.75–0.92). The cut-off score for the a* value that best differentiated individuals with severe periodontitis was 0.09, with a sensitivity of 83% and specificity of 77%. In contrast, the diagnostic accuracy of the visual inspection score for severe periodontitis was useful, with an AUC of 0.78 (95% CI: 0.67–0.87). The cut-off score for the visual inspection score was 1.25, with a sensitivity of 78% and specificity of 73%.

Table 3 presents the results of the univariable and multivariable logistic regression analyses. The a* value was associated with severe periodontitis in the univariable model (crude odds ratio [OR]: 1.90, 95% CI: 1.44–2.52). This association remained significant after adjusting for age, sex, alcohol intake, smoking status, physical activity level, overweight, and diabetes (adjusted OR: 1.86, 95% CI: 1.34–2.56). Similar results were obtained for the visual inspection score (crude OR: 4.44, 95% CI: 2.22–8.88; adjusted OR: 4.81, 95% CI: 2.06–11.24). The combined use of the TLP-AA results and the demographic and health-related variables performed well in the prediction of severe periodontitis. The multivariable model for the a* value had an AUC of 0.90, with a sensitivity of 83.3% and specificity of 77.5%. The model for the visual inspection score had an AUC of 0.88, with a sensitivity of 88.9% and specificity of 70.2%.

**Table 3:** Logistic regression models for periodontitis among Japanese workers (*n* = 347).

	Outcome: Severe periodontitis based on the CDC/AAP definition							
	Models for the a* value				Models for the visual inspection score			
	Univariable model		Multivariable model		Univariable model		Multivariable model	
	Crude OR	95% CI	Adjusted OR	95% CI	Crude OR	95% CI	Adjusted OR	95% CI
Predictor variables								
Results of the TLP-AA								
a* value	1.90	1.44–2.52	1.86	1.34–2.56				
Visual inspection score					4.44	2.22–8.88	4.81	2.06–11.24
Other variables								
Age			1.10	1.03–1.16			1.11	1.05–1.17
Men (vs. Women)			0.76	0.21–2.71			0.82	0.24–2.86
High-risk drinking			2.92	0.68–12.43			2.72	0.64–11.54
Current smoker			1.09	0.19–6.32			1.07	0.19–6.18
Low physical activity level			1.23	0.38–4.01			1.35	0.43–4.24
Overweight			5.26	1.59–17.39			4.58	1.41–14.87
Diabetes			1.30	0.12–13.64			1.35	0.14–13.14
AUC	0.83		0.90		0.78		0.88	
Sensitivity	83.3		83.3		77.8		88.9	
Specificity	76.6		77.5		73.3		70.2	
BIC	120		112		129		118	

AAP, American Academy of Periodontology; AUC, area under the receiver operating characteristic curve; BIC, Bayesian Information Criteria; CDC, Centers for Disease Control and Prevention; TLP-AA, trypsin-like peptidase activity assay

Sensitivity analyses demonstrated that the a* value showed useful diagnostic performance for severe periodontitis based on the AAP/EFP definition, with an AUC of 0.75 (95% CI: 0.68–0.82; S2 Fig). In contrast, the diagnostic accuracy of the visual inspection score for severe periodontitis based on the AAP/EFP definition was low, with an AUC of 0.69 (95% CI: 0.61–0.77; S2 Fig). Similarly, although the combined use of the visual inspection score and the demographic and health-related variables did not demonstrate good predictive performance for severe periodontitis based on the AAP/EFP definition (AUC: 0.68), the combined use of the a* value and the demographic and health-related variables performed well in the prediction of severe periodontitis based on the AAP/EFP definition (AUC: 0.82; S1 Table).

## Discussion

The key finding of this study was the high diagnostic capacity of the TLP-AA with the use of a color reader for severe periodontitis. The color reader result was associated with severe periodontitis after adjusting for other health characteristics including age, sex, alcohol intake, smoking status, physical activity level, overweight, and diabetes. Hence, the results obtained from this method may prompt an individual without self-awareness of periodontal health who does not regularly visit the dentist to seek treatment for periodontitis.

Epidemiological surveillance of severe periodontitis is important [3] because severe periodontitis increases the risks of chronic kidney disease, cardiovascular disease, and death [1, 2, 23]. However, periodontitis surveillance at the population level remains challenging because of the resources required to conduct clinical examinations for periodontitis [5, 6]. Thus, a more useful, reliable, and user-friendly method for surveillance is warranted. The TLP-AA is potentially useful in the surveillance of severe periodontitis at the population level, where clinical oral examinations are not feasible. It could enable large-scale cost-effective severe periodontitis detection in studies pertinent to periodontitis and associated systemic conditions.

In this study, the intensity of enzymatic activity in the sample was objectively assessed by measuring the color of the matrix disc in the kit. The TLP-AA results were quantified as the a* value based on the L* a* b* color system. In our preliminary study [14], the color of the matrix disc was assessed visually (i.e., visual inspection score). In the present study, we demonstrated that the a* value had a superior performance in detecting severe periodontitis than the visual inspection score. Furthermore, the a* value is objectively recorded and is not affected by an individual’s color vision.

Although the TLP-AA results have a high diagnostic performance for severe periodontitis, it has been demonstrated that the TLP-AA is not appropriate for the detection of mild and moderate periodontitis [14]. To detect mild and moderate cases, the intensity of the TLP-AA matrix disc color should be re-designed for lower trypsin concentration levels. Once this revision has been completed, investigating the application of the newly designed TLP-AA for the detection of mild and moderate periodontitis is an important next step.

In the 1980s, assessment of trypsin-like peptidase activity in subgingival plaques was introduced as a potential diagnostic marker for periodontitis [24]. However, this method is highly dependent on the sampling technique. Moreover, the obtained results are relevant to the sampled site [25]. These limitations may interfere with the broad use of this test in population-based surveillance. In contrast, we assessed the trypsin-like peptidase activity in tongue swabs; sample collection required only a few seconds and was not technically demanding, which is essential for applications in mass surveillance.

There are several biomarkers in the blood, saliva, and gingival crevicular fluid (GCF) that may be alternatives to clinical examinations. The serum immunoglobulin G antibody titers against *P*. *gingivalis* could detect periodontitis, with a sensitivity of 77%, a specificity of 59%, and an AUC of 0.71 [26]. A recent systematic review [27] reported that the interleukin-1 beta, interleukin-6, macrophage inflammatory protein-1 alpha, and matrix metalloproteinase-8 (MMP-8) levels are potential salivary biomarkers for the diagnosis of periodontitis. The combination of these four makers had a sensitivity of 81% and specificity of 78%. Other studies [28, 29] have reported that the quantification of salivary hemoglobin can be used to detect periodontitis, with a sensitivity of 72%–76% and specificity of 52%–76%. Additionally, metabolic profiling of saliva (combination of cadaverine, histidine, and 5-oxoproline) identified moderate/severe periodontitis as defined by the CDC/AAP, with an AUC of 0.88 [30]. A meta-analysis estimated the diagnostic abilities of four biomarkers in GCF: elastase, cathepsin, MMP-8, and trypsin [31]. The median sensitivity and specificity values were 75% and 81% for elastase, 73% and 67% for cathepsin, 77% and 92% for MMP-8, and 71% and 66% for trypsin, respectively. As different definitions for periodontitis were used across studies, we were unable to perform direct comparisons with the effectiveness of these periodontitis detection methods. Nevertheless, the validity of the TLP-AA used in our study for surveillance of periodontitis was comparable to that of the methods reported in other studies. Regarding sample collection, the TLP-AA-Kit is less invasive and costly than blood biomarkers, and less technically sensitive than the GCF.

The present study had several strengths. First, the sample size was adequate, as estimated based on the prevalence of severe periodontitis observed in our preliminary study [14]. Severe periodontitis was observed in 5.2% of participants in the current study population, which was comparable to that reported in our previous study (4.8%). Second, the diagnostic accuracy of the TLP-AA-Kit was tested using the FMPE data obtained by trained and calibrated examiners and by applying the CDC/AAP definition for periodontitis [20]; the CDC/AAP definition is the most commonly used definition and is considered the most appropriate definition in an epidemiological setting [32]. We also tested the diagnostic capacity of the TLP-AA using the AAP/EFP definition. The diagnostic capacity was not significantly attenuated by applying the AAP/EFP definition for periodontitis.

However, the present study had some limitations. Selection bias may have been present, as periodontal examination was performed on a voluntary basis and the study population consisted of Japanese workers from one company. Moreover, the study population mainly consisted of middle-aged adults. These factors may limit the generalizability of the study findings. The Survey of Dental Diseases (SDD), a national survey in Japan, does not involve the FMPE data. When the periodontitis definition used in the SDD 2016 [33] was applied to the current study population, the prevalence of periodontitis (defined as pocket scores of 1 or 2 for the modified Community Periodontal Index) was 53.6%. Although the observed prevalence of periodontitis in this study is comparable to that reported in the SDD 2016 (49.4%), future studies are needed to verify whether the current findings can be applied to a broader population with different working environments and age distributions.

## Conclusions

Despite the limitations of this study, we demonstrated that objectively assessed TLP-AA results have high diagnostic capacity for the detection of severe periodontitis in population-based surveillance.

## Supporting information

S1 Fig Trypsin-like peptidase activity assay kit. The kit is presented (a) before and (b) after testing. (c) The a* value of the matrix disc color is measured using an FD-7 Color Reader.
(TIF)

S2 Fig Receiver operating characteristic curves of TLP-AA results for severe periodontitis based on the AAP/EFP definition. Receiver operating characteristic curves of the TLP-AA results for severe periodontitis as defined by the AAP/EFP. AAP, American Academy of Periodontology, EFP, European Federation of Periodontology, TLP-AA, trypsin-like peptidase activity assay.
(TIF)

S1 Table Logistic regression models for periodontitis based on the AAP/EFP definition among Japanese workers (*n* = 347). (DOCX)
