# Supplementary figures and images for: Evaluation of the ability of the trypsin-like peptidase activity assay to detect severe periodontitis
Source: PLoS One. 2021 Aug 20;16(8):e0256538. doi: 10.1371/journal.pone.0256538 (PMC8378714; doi:10.1371/journal.pone.0256538)

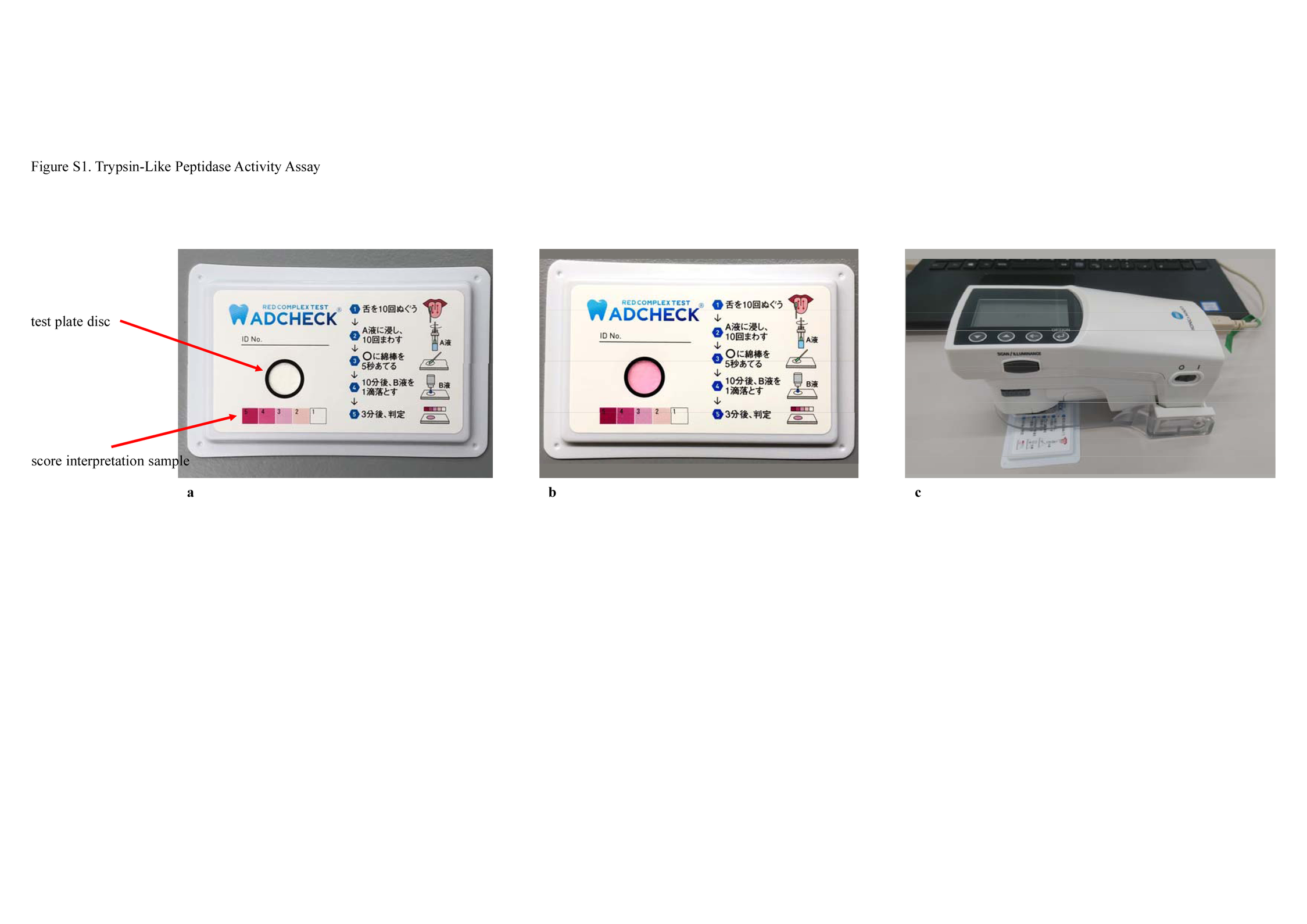

Supplement: S1 Fig — The kit is presented (a) before and (b) after testing. (c) The a* value of the matrix disc color is measured using an FD-7 Color Reader. (TIF) [file pone.0256538.s001.tif]

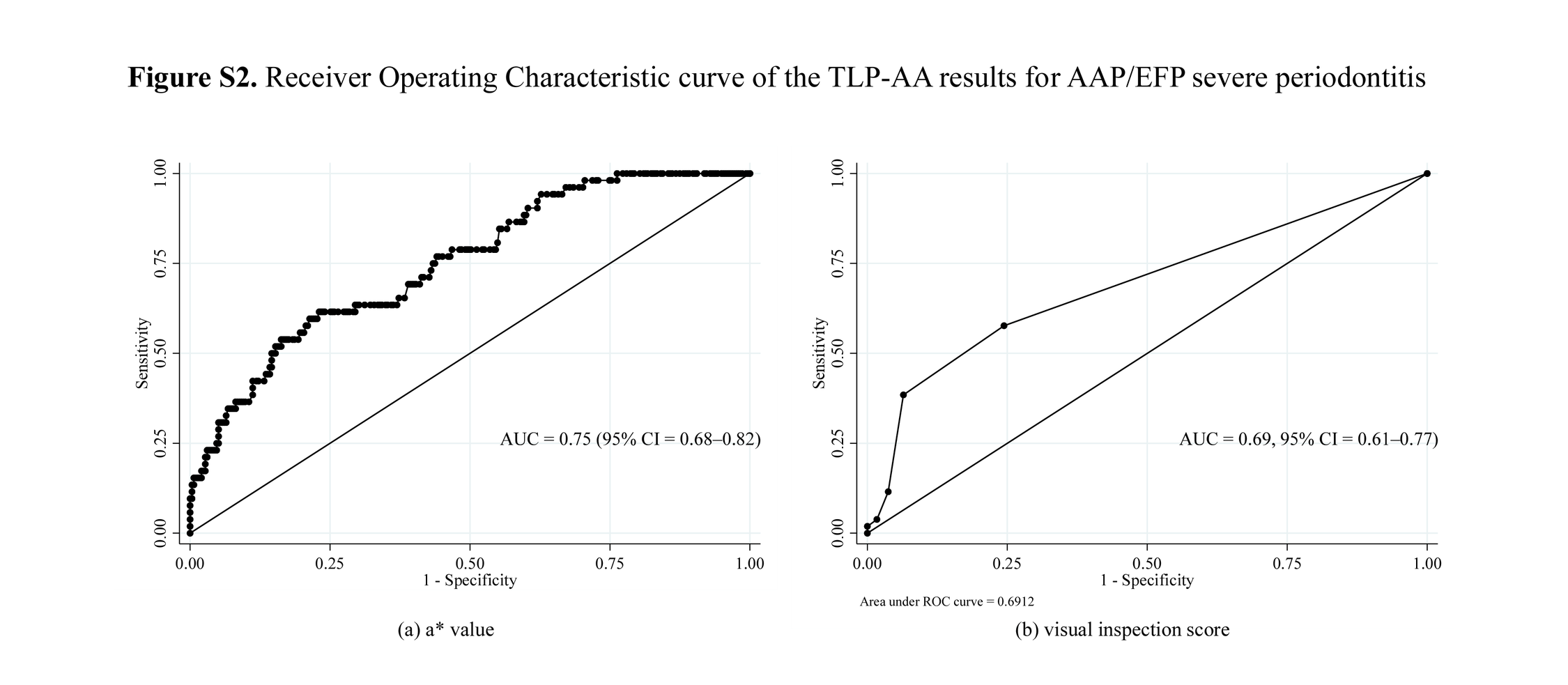

Supplement: S2 Fig — Receiver operating characteristic curves of the TLP-AA results for severe periodontitis as defined by the AAP/EFP. AAP, American Academy of Periodontology, EFP, European Federation of Periodontology, TLP-AA, trypsin-like peptidase activity assay. (TIF) [file pone.0256538.s002.tif]
