# Supplementary material for: Evaluation of the ability of the trypsin-like peptidase activity assay to detect severe periodontitis
Source: PLoS One. 2021 Aug 20;16(8):e0256538. doi: 10.1371/journal.pone.0256538 (PMC8378714; doi:10.1371/journal.pone.0256538)
Supplement: S1 Table — (DOCX) [file pone.0256538.s003.docx]

| **S1 Table. Logistic regression models for periodontitis based on the AAP/EFP definition among Japanese workers (*n*=347)** | | | | | | | | |
| --- | --- | --- | --- | --- | --- | --- | --- | --- |
|  | Outcome: Severe periodontitis based on the AAP/EFP definition | | | | | | | |
|  | Models for the a* value | | | | Models for the visual inspection score | | | |
|  | Univariable model | | Multivariable model | | Univariable model | | Multivariable model | |
|  | Crude OR | 95% CI | Adjusted OR | 95% CI | Crude OR | 95% CI | Adjusted OR | 95% CI |
| Predictor variables |  |  |  |  |  |  |  |  |
| Results of the TLP-AA |  |  |  |  |  |  |  |  |
| a* value | 1.99 | 1.57–2.52 | 1.98 | 1.52–2.58 |  |  |  |  |
| Visual inspection score |  |  |  |  | 2.07 | 1.21–3.53 | 1.82 | 1.01–3.3 |
| Other variables |  |  |  |  |  |  |  |  |
| Age |  |  | 1.06 | 1.03–1.1 |  |  | 1.07 | 1.04–1.1 |
| Men (vs. Women) |  |  | 1.41 | 0.68–2.92 |  |  | 1.35 | 0.67–2.7 |
| High-risk drinking |  |  | 1.39 | 0.49–3.93 |  |  | 1.60 | 0.62–4.17 |
| Current smoker |  |  | 1.20 | 0.44–3.32 |  |  | 1.28 | 0.48–3.38 |
| Low physical activity level |  |  | 1.07 | 0.53–2.15 |  |  | 1.19 | 0.62–2.28 |
| Overweight |  |  | 2.39 | 1.06–5.36 |  |  | 1.91 | 0.91–4.04 |
| Diabetes |  |  | 0.79 | 0.13–4.83 |  |  | 0.72 | 0.14–3.85 |
|  |  |  |  |  |  |  |  |  |
| AUC | 0.75 |  | 0.82 |  | 0.60 |  | 0.68 |  |
| Sensitivity | 61.5 |  | 76.9 |  | 44.2 |  | 67.3 |  |
| Specificity | 77.0 |  | 75.9 |  | 73.2 |  | 70.9 |  |
| BIC | 254 |  | 240 |  | 291 |  | 272 |  |
|  |  |  |  |  |  |  |  |  |
| AAP, American Academy of Periodontology; AUC, area under the receiver operating characteristic curve; BIC, Bayesian Information Criteria; EFP, European Federation of Periodontology; TLP-AA, trypsin-like peptidase activity assay | | | | | | | | |
